# Maternal Pregestational Body Mass Index and Delivery Outcomes: A Retrospective Study in a Tertiary High‐Income Setting

**DOI:** 10.1155/jnme/6251114

**Published:** 2026-06-29

**Authors:** Francesca Parisi, Ottavio Cassardo, Lucrezia Viscioni, Vittorio Parodi, Marta Tondo, Benedetta Gallicola, Chiara Lubrano, Ilma Floriana Carbone, Emanuela Taricco, Enrico Iurlaro, Irene Cetin

**Affiliations:** ^1^ Department of Biomedical and Clinical Sciences, University of Milan, Milan, Italy, unimi.it; ^2^ Unit of Obstetrics, Foundation IRCCS Ca’ Granda Ospedale Maggiore Policlinico, Milan, Italy, policlinico.mi.it; ^3^ Nutritional Sciences, Doctoral Programme (PhD), University of Milan, Milan, 20157, Italy, unimi.it; ^4^ Department of Clinical Sciences and Community Health, Dipartimento di Eccellenza 2023-2027, Università Degli Studi di Milano, Milan, Italy, unimi.it

**Keywords:** cesarean delivery, maternal obesity, neonatal morbidity, overweight, postpartum hemorrhage, pregnancy outcomes, underweight

## Abstract

**Background:**

Maternal overweight (OW) and obesity are increasingly prevalent worldwide and have been linked to adverse pregnancy and delivery outcomes. This study aims to evaluate maternal and neonatal outcomes in pregnancies stratified according to pregestational body mass index (BMI) in a tertiary care setting in Italy.

**Methods:**

This retrospective study included all singleton live births between 2022 and 2024 at the IRCCS Foundation Ca’ Granda Ospedale Maggiore Policlinico, Milan, Italy. Women were classified into four groups based on pregestational BMI: (1) underweight (UW, BMI < 18.5 kg/m^2^, *n* = 1796); (2) normal weight (NW, BMI 18.5–24.9 kg/m^2^, *n* = 11,710); (3) OW (BMI ≥ 25.0 kg/m^2^, *n* = 2313); and (4) obese (OB, BMI ≥ 30.0 kg/m^2^, *n* = 976). Delivery outcomes were compared among groups using binary logistic regression and generalized linear models adjusted for confounding factors.

**Results:**

Inclusion criteria were met by a population of 17,813 singleton pregnancies. Multivariate models confirmed that OB women were independently associated with virtually all adverse delivery outcomes, including higher odds of preterm birth (aOR 1.48), labor induction (aOR 1.69), emergency cesarean delivery (aOR 1.42), postpartum hemorrhage (aOR 1.40), APGAR score at 5 min lower than 6 (aOR 2.77), and admission to neonatal intensive care unit (aOR 2.05) compared to NW controls. OW women were at higher risk of emergency cesarean delivery and postpartum hemorrhage, while UW women showed largely comparable outcomes to NW women, with the sole exception of reduced birthweights and higher odds of episiotomy use.

**Conclusions:**

Maternal OW and OB are associated with increased maternal and neonatal morbidity, highlighting the complex obstetric challenges in this population. These findings underscore the need for a BMI‐tailored obstetric care and preventive public health strategies targeting women of reproductive age.

## 1. Introduction

Modern health care has significantly enhanced the safety of pregnancy and childbirth for women living in high‐income countries. In the Global North, the maternal mortality rate has currently reached very low values of less than 10 per 10,000 births, while stillbirth and late fetal death rates range between four and six per 1000 births [1]. Nevertheless, changes in nutritional habits and the increasing global prevalence of overweight (OW) and obesity among women of reproductive age represent a major public health concern, particularly in the context of reproduction and pregnancy outcomes.

Roughly 39 million pregnancies per year are complicated by maternal obesity worldwide, with an estimated prevalence of OW and obesity in pregnancy over 60% in some countries [2–7]. Overall, OW and obesity are associated with an increased risk of virtually all pregnancy‐related complications, representing an independent key risk factor for gestational diabetes mellitus (GDM), hypertensive disorders, preterm birth, labor complications, and perinatal and maternal mortality [8–11]. Several studies additionally showed higher rates of stillbirth in pregnancies complicated by OW or obesity, with an additional double risk in case of associated excessive gestational weight gain beyond 39 gestational weeks [12]. In particular, a systematic review and meta‐analysis encompassing over 16,000 stillbirths further concluded increased odds of stillbirth by 24% (OR 1.24, 95% CI 1.18–1.30) for each 5 kg/m^2^ increase in body mass index (BMI) above the ideal range [13]. Obese (OB) women are also more likely to require labor induction, further facing a substantially higher risk of induction failure and emergency cesarean delivery compared to women with a normal BMI [4, 14–17]. Finally, maternal obesity is reported to increase the risk of surgical complications during delivery and represents a major risk factor for postpartum hemorrhage (PPH) [16–18]. On the other hand, pre‐pregnancy underweight (UW) as well as insufficient gestational weight gain have been associated with preterm delivery and small for gestational age (SGA) babies [19]. UW also significantly impacts the risk of perineal trauma, independently of GWG, with lasting implications for pelvic floor function and quality of life [20, 21].

The underlying pathophysiological mechanisms remain to be fully elucidated. In OB pregnancy, chronic low‐grade inflammation and oxidative stress have been described at the maternal–placental interface, together with increased risk of iatrogenic preterm delivery due to higher risks of pregnancy complications [22]. Histological and transcriptomic studies have also shown elevated placental inflammatory markers, inflammasome activation (e.g., IL‐1β), and vascular malperfusion in OB pregnancies [18, 23–25]. This proinflammatory placental environment may trigger premature activation of labor pathways or contribute to placental dysfunction, ultimately increasing the risk of both spontaneous and medically indicated preterm birth [23, 26]. Nevertheless, further complicating the pathophysiological picture, the relationship between maternal BMI and preterm birth is not always simply linear. A recent case–control study by Hunter et al. suggests an “obesity paradox” in which, particularly for women of lower socioeconomic status, higher BMI may be associated with a decreased risk of preterm birth. This finding underscores the influence of social determinants of health and highlights the need for a nuanced understanding of BMI’s role in preterm birth [24].

The aim of the present study is to provide up‐to‐date real‐world evidence on the associations between pre‐pregnancy BMI and delivery outcomes in a high‐resource obstetric environment.

## 2. Materials and Methods

### 2.1. Study Design and Population

This is a retrospective, observational, single‐center, nonprofit study conducted at the Obstetrics Unit of the tertiary care hospital, IRCCS Foundation Ca’ Granda Ospedale Maggiore Policlinico, Milan, Italy. The study was approved by the Institutional Review Board (IRB), and all included patients signed a written informed consent (Ethic Committee Protocol Number 6654). Data were retrospectively extracted from digital medical records of the CEDAP system (Certificato di Assistenza al Parto—Certificate of Assistance at Birth), a regional digital registry of birth‐related medical data, and validated against hospital birth registries and electronic medical records. All data were thoroughly anonymized before the analysis. The study population consisted of all women who delivered at our institution between 2022 and 2024, with the only exclusion criteria being multiple pregnancies, women undergoing induction of abortive labor before 23 weeks of gestation, and missing pregestational BMI data. Cases of intrauterine fetal death are reported in the table, but no multivariable analyses were performed for this outcome due to the limited number of events The cohort was divided into four groups: (1) UW (BMI < 18.5 kg/m^2^); (2) normal weight (NW, BMI 18.5–24.9 kg/m^2^); (3) OW (BMI 25.0–29.9 kg/m^2^); and (4) OB (BMI ≥ 30.0 kg/m^2^).

### 2.2. Data Collection

Pregnancy and delivery data were collected, including maternal, labor, and neonatal parameters at birth. As an exposure variable, BMI was calculated using the formula weight (kg)/height (m^2^), based on the pregestational weight reported by the woman and height as measured by the healthcare providers at the first antenatal visit. As covariates, maternal age, parity (nulliparous versus multiparous), ethnicity (Caucasian versus non‐Caucasian), history of previous cesarean deliveries (yes versus no), mode of conception (spontaneous versus assisted reproductive technology), use of epidural analgesia (yes versus no), and neonatal sex (male versus female) were included in the analysis. Pregnancy complications were assessed as a composite parameter that includes hypertensive disorders of pregnancy and GDM (yes versus no). As outcome measures, pregnancy and delivery data included gestational age at delivery, labor induction (yes versus no), mode of delivery (spontaneous vaginal, vacuum‐assisted vaginal birth, planned or emergency cesarean delivery), episiotomy (yes versus no), severe perineal tears (yes versus no, defined as perineal tears of third to fourth degree), and PPH (defined as blood loss higher than 500 mL after vaginal delivery or 1000 mL after cesarean delivery). Neonatal outcomes included birthweight, Apgar scores at 5 minutes, and admission to the neonatal intensive care unit (NICU).

### 2.3. Statistical Analysis

The large number of subjects and variables involved suggests a high likelihood that missing data were random, making a complete case analysis more appropriate for this population‐wide study. Continuous variables are expressed as medians and ranges, whereas categorical variables are expressed as absolute and relative frequencies. Comparisons among the four groups were conducted using the chi‐square test for categorical variables and the Kruskal–Wallis test or one‐way ANOVA for continuous variables, as appropriate. Binary logistic regression and generalized linear models adjusted for potential confounders (maternal age, ethnicity, conception mode, previous cesarean delivery, pregnancy complications, fetal sex, induction of labor, epidural analgesia, gestational age at delivery, and birthweight) were conducted to analyze the associations between BMI‐based groups (independent variable) and delivery and neonatal outcomes (dependent variables), thus providing odds ratios (ORs), beta values, and related confidence intervals (CIs). All statistical analyses were performed using IBM SPSS Statistics, Version 29.0 (IBM Corp., Armonk, NY, USA). Statistical significance was set at *p* < 0.05.

## 3. Results

During the study period, 18,221 singleton pregnancies were delivered at the IRCCS Foundation Ca’ Granda Ospedale Maggiore Policlinico, Milan, Italy. Multiple pregnancies (*n* = 1015), women with intrauterine fetal demise undergoing labor induction (*n* = 32), and missing pregestational BMI values (*n* = 380) were excluded from further analyses. Therefore, the total included population counted 16,795 women, including 1796 (10.7%) in the UW group, 11,710 (69.7%) in the NW group, 2313 (13.8%) in the OW group, and 976 (5.8%) in the OB group. Table 1 shows antepartum data of the included population with comparisons among the study groups.

**TABLE 1:** Antepartum data with comparisons among the study groups.

	UW (*n* = 1796, 10.7%)	NW (*n* = 11,710, 69.7%)	OW (*n* = 2313, 13.8%)	OB (*n* = 976, 5.8%)	*p*‐value
Maternal age, years, median (range)	35 (17–51)	35.0 (15–57)	35.0 (16–54)	34 (17–53)	< 0.001
Non‐Caucasian ethnicity, *n* (%)	259 (14.4)	2067 (17.7)	737 (31.9)	346 (35.5)	< 0.001
Nulliparous, *n* (%)	1120 (62.4)	7344 (62.7)	1210 (52.3)	470 (48.2)	< 0.001
Prior cesarean delivery, *n* (%)	137 (7.6)	843 (7.2)	268 (11.6)	140 (13.3)	< 0.001
ART, *n* (%)	181 (10.1)	1259 (10.8)	220 (9.5)	75 (7.7)	0.01
Pregnancy complications	260 (14.5)	1780 (15.2)	453 (19.6)	218 (22.3)	< 0.01
Intrauterine fetal demise	1 (0.1)	14 (0.1)	6 (0.3)	2 (0.2)	0.26

*Note:* Comparisons among the four groups were conducted using the Chi‐square test for categorical variables and the Kruskal–Wallis test or one‐way ANOVA for continuous variables, as appropriate. Pregnancy complications represent a composite parameter that includes hypertensive disorders of pregnancy and gestational diabetes mellitus. ART: assisted reproductive technology, NW: normal weight, OW: overweight, OB: obese, UW: underweight.

Table 2 presents intrapartum data and delivery outcomes across the study groups. With the sole exception of severe perineal tears and APGAR scores lower than 7, which occurred at comparable rates across groups, significant differences were observed for all study outcomes. In particular, the OB group exhibited higher rates of preterm birth, labor induction, cesarean delivery (both planned and emergency), and PPH compared with the reference NW group. Conversely, the same group showed lower rates of vaginal and vacuum‐assisted deliveries, epidural, and episiotomy use compared to NW controls. Regarding neonatal outcomes, significant differences were observed in birthweight and NICU admission, with these outcomes consistently higher in the OB group.

**TABLE 2:** Intrapartum data with comparisons across the study groups.

Characteristic	UW (*n* = 1796, 10.7%)	NW (*n* = 11,710, 69.7%)	OW (*n* = 2313, 13.8%)	OB (*n* = 976, 5.8%)	*p*‐value
Gestational age, weeks, median (range)	39 + 2 (24–42)	39 + 2 (22–41)	39 + 1 (24–41)	39 + 2 (23–41)	< 0.001
Preterm birth, *N* (%)	139 (7.7)	659 (5.6)	163 (7.0)	84 (8.6)	< 0.001
Induction of labor, *n* (%)	596 (46.0)	4064 (48.6)	773 (49.1)	325 (57.4)	< 0.01
Epidural analgesia *n* (%)	1014 (56.5)	6896 (58.0)	1185 (51.2)	439 (45.0)	< 0.001
Vaginal delivery, *n* (%)	1032 (57.5)	6548 (55.9)	1144 (49.5)	455 (46.6)	< 0.001
Vacuum‐assisted delivery, *n* (%)	128 (7.1)	806 (6.9)	117 (5.1)	30 (3.1)	< 0.001
Cesarean delivery, *n* (%)	636 (35.4)	4356 (37.2)	1052 (45.5)	491 (50.3)	< 0.001
Emergency cesarean delivery *n* (%)	186 (10.4)	1453 (12.4)	390 (16.9)	149 (15.3)	< 0.001
Episiotomy, *n* (%)	434 (24.2)	2690 (23.0)	402 (17.4)	110 (11.3)	< 0.001
Severe perineal tears, *n* (%)	5 (0.3)	40 (0.3)	7 (0.3)	4 (0.4)	0.93
Postpartum hemorrhage, *n* (%)	153 (8.5)	1160 (9.9)	276 (11.9)	119 (12.2)	< 0.001
Birthweight, grams, median (range)	3130 (680–4970)	3260 (490–5000)	3320 (450–4570)	3340 (470–4730)	< 0.001
Male fetus, *n* (%)	897 (49.9)	6039 (51.6)	1186 (51.3)	488 (50.0)	0.50
Apgar score < 7 at 5 min, *n* (%)	9 (0.5)	37 (0.3)	11 (0.5)	7 (0.7)	0.14
NICU admission, *n* (%)	40 (2.2)	199 (1.7)	53 (2.3)	37 (3.8)	< 0.001

*Note:* Comparisons among the four groups were conducted using the chi‐square test for categorical variables and the Kruskal–Wallis test or one‐way ANOVA for continuous variables, as appropriate. NW: normal weight, OW: overweight, OB: obese, UW: underweight.

**FIGURE 1:**
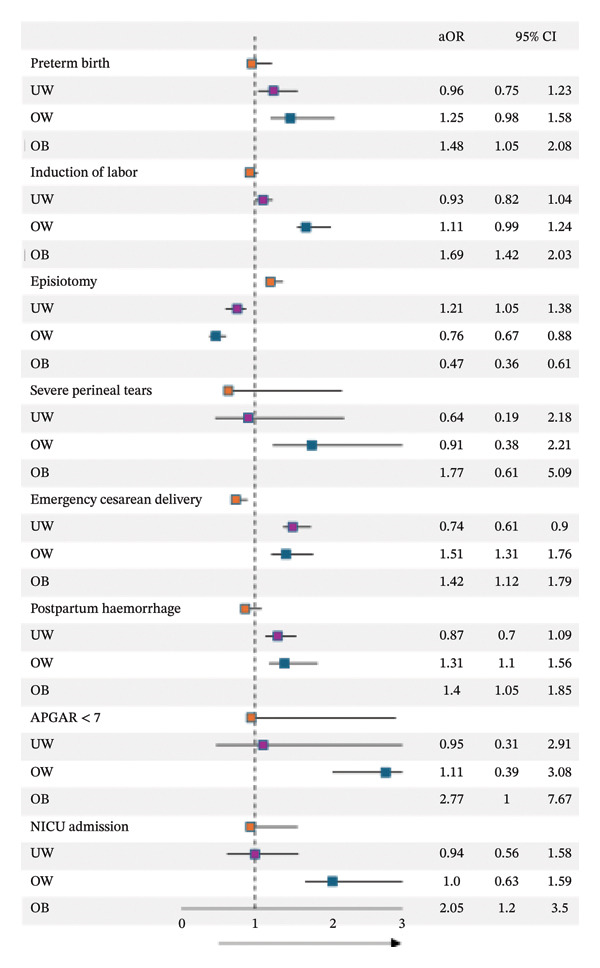
Association between maternal pre‐pregnancy BMI categories and adverse maternal and neonatal outcomes. Forest plot showing adjusted odds ratios (aORs) and 95% confidence intervals (CIs) for selected maternal and neonatal outcomes according to maternal pre‐pregnancy body mass index (BMI) categories. Normal‐weight women served as the reference group. Underweight (UW), overweight (OW), and obese (OB) categories are shown for each outcome. The vertical dashed line indicates the null value (aOR = 1). Adjusted odds ratios were derived from multivariable logistic regression models.

The results of the logistic regression models are presented in Table 3. The OW and OB groups were confirmed to be independently associated with several adverse delivery outcomes. Specifically, both groups showed a higher likelihood of emergency cesarean delivery and PPH compared with NW women. The OB group also showed a higher risk of preterm birth (< 37 weeks) and induction of labor compared to controls. With respect to neonatal outcomes, neonates born to OB women showed higher odds of APGAR scores lower than 7 at 5 minutes, as well as an increase in NICU admission compared to the NW group. On the other hand, the UW women showed a reduced likelihood of emergency cesarean delivery and a significant increase in episiotomy rate compared to the reference group (Figure 1).

**TABLE 3:** Results from logistic regression models for the associations between BMI‐based groups and delivery outcomes.

	NW	UW aOR (95% CI)	OW aOR (95% CI)	OB aOR (95% CI)
Preterm birth	Ref	0.96 (0.75–1.23)	1.25 (0.98–1.58)	1.48 (1.05–2.08)^∗^
Induction of labor	Ref	0.93 (0.82–1.04)	1.11 (0.99–1.24)	1.69 (1.42–2.03)^∗∗∗^
Episiotomy	Ref	1.21 (1.05–1.38)^∗∗^	0.76 (0.67–0.88)^∗∗∗^	0.47 (0.36–0.61)^∗∗∗^
Severe perineal tears	Ref	0.64 (0.19–2.18)	0.91 (0.38–2.21)	1.77 (0.61–5.09)
Emergency cesarean delivery	Ref	0.74 (0.61–0.90)^∗∗^	1.51 (1.31–1.76)^∗∗∗^	1.42 (1.12–1.79)^∗∗∗^
Postpartum hemorrhage	Ref	0.87 (0.70–1.09)	1.31 (1.10–1.56)^∗∗^	1.40 (1.05–1.85)^∗^
APGAR < 7	Ref	0.95 (0.31–2.91)	1.11 (0.39–3.08)	2.77 (1.00–7.67)^∗^
NICU admission	Ref	0.94 (0.56–1.58)	1.00 (0.63–1.59)	2.05 (1.20–3.50)^∗∗^

*Note:* Binary logistic regression models were employed to evaluate the associations between BMI‐based groups and delivery outcomes, with the NW group as reference. The model for preterm birth was adjusted for maternal age, ethnicity, parity, prior cesarean delivery, ART conception, fetal sex, birthweight, and pregnancy complications. Further adjustment for gestational age at delivery, induction of labor, epidural use, and delivery mode was included for the remaining models. Postpartum hemorrhage was defined as blood loss higher than 500 mL after vaginal delivery or 1000 mL after cesarean delivery. ^∗^ *p* < 0.05; ^∗∗^ *p* < 0.01, ^∗∗∗^ *p* < 0.001.

**FIGURE 2:**
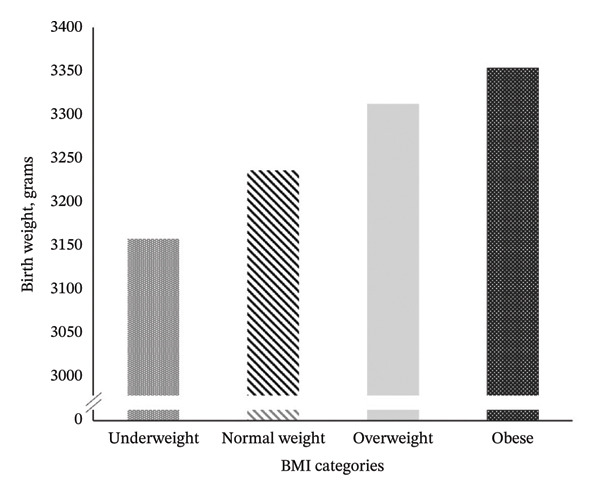
Association between maternal pre‐pregnancy body mass index (BMI) categories and normalized fetal weight at 38 + 6 weeks. Mean fetal birthweight (grams) stratified by maternal pre‐pregnancy BMI categories (underweight, normal weight, overweight, and obese). To account for differences in gestational age at delivery, fetal birthweights were normalized to 38 + 6 weeks using a gestational age–adjusted linear model. Individual birthweights were recalculated as the estimated weight at 38 + 6 weeks, allowing comparison across maternal BMI categories independently of delivery timing. A significant positive gradient is observed, demonstrating that birthweight progressively increases across higher maternal BMI categories.

**FIGURE 3:**
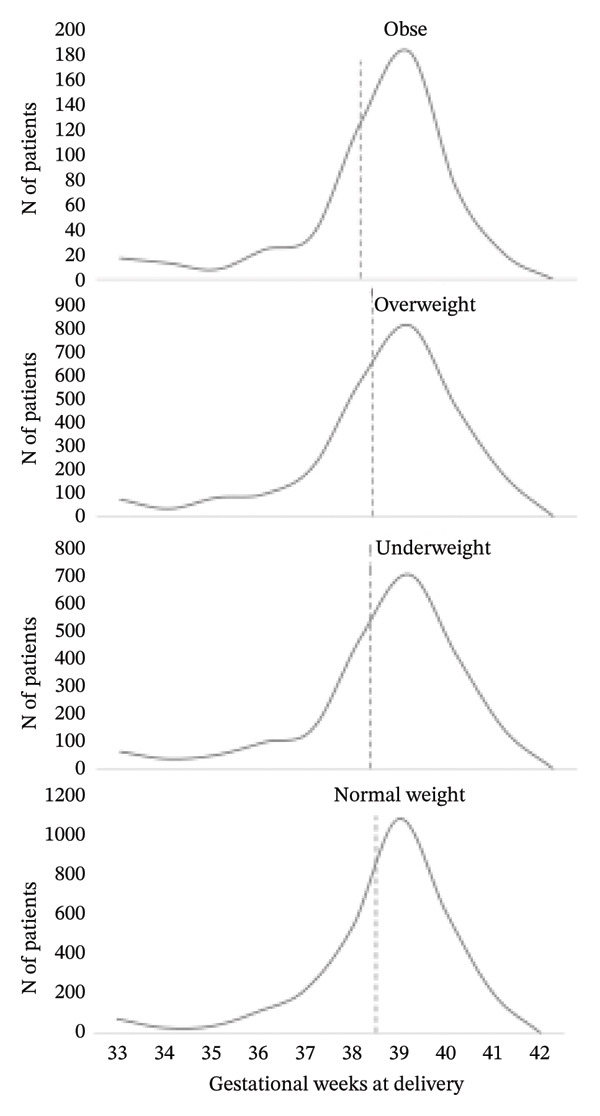
Gestational age distribution in obese versus normal weight pregnancies. Kernel density plots of gestational age at delivery stratified by maternal BMI category. Normal‐weight women show the highest concentration of deliveries around term, with a mean gestational age of 38.54 weeks. In obese women, the distribution is wider and shifted toward earlier gestational ages, with a lower mean value (38.12 weeks). Underweight and overweight women show intermediate distributions, with mean gestational ages of 38.40 and 38.36 weeks, respectively. Overall, increasing deviation from normal BMI is associated with greater dispersion and a tendency toward earlier delivery.

Lastly, generalized linear models showed significant associations between BMI‐based groups, birthweight (UW: *β* = −81.7 g [95% CI: −100.1; −63.3], *p* < 0.001; OW: *β* = 49.0 g [95% CI: 32.0; 65.6], *p* < 0.001; OB: *β* = 88.6 g [95% CI: 64.2; 113.0], *p* < 0.001) (Figure 2) and gestational age at delivery (UW: *β* = 0.08 weeks [95% CI: 0.01; 0.14], *p* < 0.05; OW: *β* = −0.18 weeks [95% CI: −0.24; −0.12], *p* < 0.001; OB: *β* = −0.40 weeks [95% CI: −0.49; −0.32], *p* < 0.001) (Figure 3).

## 4. Discussion

In this study, significant associations emerged between pregestational BMI categories and maternal as well as neonatal outcomes at delivery within a large cohort of singleton pregnancies resulting in live births at a tertiary care center in Italy. In multivariable‐adjusted models, women in the OB group demonstrated markedly increased risks relative to the control group, including a higher risk of preterm birth, induction of labor, emergency cesarean delivery, and PPH, alongside a reduced likelihood of episiotomy. OB women also exhibited elevated risks of low Apgar scores (< 7) and a twofold increase in NICU admission. Women in the OW group showed increased risk of emergency cesarean delivery and PPH, accompanied by a reduced frequency of episiotomy compared with the NW reference group. By contrast, women in the UW group demonstrated a lower risk of emergency cesarean delivery, an increased likelihood of episiotomy, and a significantly lower birthweight relative to the reference group. These observations reaffirm the complex obstetric challenges posed by maternal deranged metabolism, supporting the need for tailored obstetric and neonatal care according to maternal nutritional status.

### 4.1. Maternal and Pregnancy Characteristics

In the present study, OW and OB women exhibited several baseline characteristics that differed from those of the NW controls. They were more likely to be non‐Caucasian, multiparous, and to have a history of prior cesarean delivery. These findings are consistent with existing evidence showing a higher prevalence of obesity in specific ethnic groups [3, 20] and an association between obesity and previous cesarean delivery, which may reflect underlying obstetric risk profiles or a reduced likelihood of achieving a successful vaginal birth after cesarean in this population [4, 17]. In line with previous data [25, 27], maternal age was slightly, but significantly lower among OB women—a trend that may reflect broader demographic and sociocultural patterns. In particular, women with higher socioeconomic status, who are generally less likely to be OB in the Italian context, also tend to defer childbearing to later ages, contributing to the observed age differences [28–30]. Notably, the OB and OW groups also showed higher rates of pregnancy complications, a composite parameter that includes hypertensive disorders of pregnancy and GDM. This observation highlights the well‐known increased baseline burden of maternal and fetal complications in OW and OB women [28–30].

Although the median gestational age at delivery differed only slightly across BMI‐based groups, multivariable linear modeling demonstrated a statistically significant association between maternal obesity and reduced gestational length, further confirmed by a significantly higher risk of preterm delivery (aOR 1.48, 95% CI: 1.05–2.08) solely in this group compared to controls. In line with our result, a large Mendelian‐randomization study further supports a causal relationship between obesity and preterm birth, reporting an OR of 1.50 per standard deviation increase in maternal BMI [11].

### 4.2. Intrapartum Outcomes

Delivery outcomes were less favorable among OB and OW women, as highlighted by higher rates of labor induction, cesarean delivery (both overall and emergency), and PPH. Logistic regression models confirmed that these groups were also independently associated with increased likelihood of PPH and emergency cesarean delivery among women admitted to vaginal delivery. These associations mirror previous findings from multiple large‐scale studies, observing similar trends in delivery interventions among OB pregnant women [14, 31]. In our analysis, the increased likelihood of emergency cesarean delivery persisted after adjustment for multiple confounders, including labor induction and pregnancy complications, suggesting that maternal obesity itself may predispose individuals to adverse intrapartum outcomes through pathophysiological mechanisms that extend beyond the mere accumulation of comorbidities [2]. Specifically, through systemic low‐grade inflammation, endothelial dysfunction, and prothrombotic state, obesity appears to be intrinsically linked to impaired uteroplacental perfusion [32]. This compromised vascular environment may, in turn, set the trajectory of labor toward placental and fetal malperfusion and suboptimal uterine contractility, thus explaining the detected increased risks of cesarean delivery and PPH [33, 34]. Conversely, rates of instrumental delivery (vacuum extraction) and episiotomy were lower among OB and OW women. This pattern may reflect clinicians’ reluctance to perform assisted vaginal delivery in OB patients due to technical challenges or perceived risks of shoulder dystocia, as previously suggested by Kominiarek et al. [34]. Although lower episiotomy rates might be interpreted as favorable, they may also indicate a shift in obstetric practice patterns, potentially favoring cesarean delivery over assisted vaginal birth in these populations [35].

### 4.3. Neonatal Outcomes

With regard to neonatal outcomes, infants of OW and OB mothers had significantly higher birthweights, a finding aligned with well‐documented links between maternal obesity and fetal macrosomia [35]. Although the overall median birthweight remained within normal ranges, an increased fetal size poses clinical challenges during labor and is a known contributor to intrapartum complications, such as prolonged labor, shoulder dystocia, and cesarean delivery [9]. Maternal obesity was associated with an increased risk of neonatal Apgar scores below 7 at 5 min and NICU admission, highlighting serious concerns about perinatal compromise [36–38]. These findings are consistent with prior studies linking maternal obesity to neonatal respiratory morbidity, possibly due to delayed fetal lung maturity and altered placental function [39–41].

### 4.4. UW Patients

Compared with controls, the UW group exhibited a lower median birthweight (3130 g vs. 3260 g), a finding confirmed in multivariable‐adjusted models and consistent with robust evidence linking maternal undernutrition to reduced gestational length and impaired fetal and placental growth [18, 23, 37, 38]. As birthweight remains the primary proxy for future health outcomes and disease risk in the offspring, this result underscores the importance of timely identification and targeted intervention in this high‐risk group to improve maternal nutritional status and long‐term health trajectories. Conversely, for the remaining delivery outcomes, the UW and NW groups showed comparable risks, including similar rates of preterm birth, PPH, and adverse neonatal outcomes. The significantly increased likelihood of episiotomy aligns with previous evidence of increased perineal injury in this group of patients (ref scritte già nella intro), suggesting a more careful approach in managing vaginal deliveries within this subpopulation.

It is plausible that the favorable outcomes observed among UW women may be partially influenced by selection bias within this group. Specifically, the UW category may include a subset of women characterized by optimal pre‐pregnancy fitness, higher socioeconomic and educational status, and adequate gestational weight gain. These factors could mitigate the adverse effects typically associated with low pre‐pregnancy BMI, thereby attenuating the overall risk profile in this population [19].

### 4.5. Strengths and Limitations

A key strength of this study is the large sample size with a population‐scale design, allowing for robust comparisons among groups, accounting for potential confounders. The study population—women delivering in a high‐volume tertiary care center—also enhances the generalizability to similar clinical settings. Nonetheless, the study has a few limitations. Information on smoking status, gestational weight gain, and educational level was not available. Furthermore, the retrospective design of the study precludes causal inferences. Also, BMI categorization did not account for the gradations of obesity (e.g., Class I vs. Class III), which may have masked more nuanced dose–response relationships. Lastly, pre‐pregnancy BMI was calculated based on self‐reported maternal weight, potentially leading to misclassification across BMI categories. These limitations may have introduced residual confounding and attenuated or obscured the true magnitude and gradient of the associations observed between BMI and maternal and neonatal outcomes.

## 5. Conclusions

In this large cohort of singleton pregnancies, pregestational BMI was independently associated with several maternal and neonatal outcomes at delivery. Obesity was linked to higher risks of preterm birth, labor induction, emergency cesarean delivery, PPH, and adverse neonatal outcomes, including low Apgar scores and NICU admission. OW women also showed increased risks of emergency cesarean delivery and PPH, while UW women were characterized by lower birthweight and a higher likelihood of episiotomy, with otherwise comparable outcomes to NW women. These findings highlight the significant impact of maternal BMI on intrapartum management and perinatal outcomes in a tertiary care setting. From a public health perspective, these data underscore the importance of obesity prevention and management in reproductive‐aged women. Interventions targeting weight loss before conception and gestational weight gain have demonstrated significant benefits, including reduced gestational complications and improved neonatal outcomes [42, 43]. Future research should explore the underlying mechanisms linking maternal obesity to adverse perinatal outcomes, including the role of inflammation, insulin resistance, and placental dysfunction [44]. Prospective cohort studies incorporating metabolic profiles and detailed obstetric histories may help clarify these associations and guide more individualized care strategies.

## Funding

This paper was partially funded by the Italian Ministry of Health. Open access publishing facilitated by Universita degli Studi di Milano, as part of the Wiley ‐ CRUI‐CARE agreement.

## Conflicts of Interest

The authors declare no conflicts of interest.

## Data Availability

The data supporting the findings of this study are available from the corresponding author upon reasonable request.
